# Supplementing L-Citrulline Can Extend Lifespan in *C. elegans* and Attenuate the Development of Aging-Related Impairments of Glucose Tolerance and Intestinal Barrier in Mice

**DOI:** 10.3390/biom13111579

**Published:** 2023-10-26

**Authors:** Dragana Rajcic, Franziska Kromm, Angélica Hernández-Arriaga, Annette Brandt, Anja Baumann, Raphaela Staltner, Amélia Camarinha-Silva, Ina Bergheim

**Affiliations:** 1 Department of Nutritional Sciences, Molecular Nutritional Science, University of Vienna, 1090 Vienna, Austria; 2 Institute of Animal Science, University of Hohenheim, 70593 Stuttgart, Germanyamelia.silva@uni-hohenheim.de (A.C.-S.)

**Keywords:** microbiota, *C. elegans*, senescence, nitric oxide, lifespan

## Abstract

L-Citrulline (L-Cit) is discussed to possess a protective effect on intestinal barrier dysfunction but also to diminish aging-associated degenerative processes. Here, the effects of L-Cit on lifespan were assessed in *C. elegans*, while the effects of L-Cit on aging-associated decline were determined in C57BL/6J mice. For lifespan analysis, *C. elegans* were treated with ±5 mM L-Cit. Twelve-month-old male C57BL/6J mice (*n* = 8–10/group) fed a standard chow diet received drinking water ± 2.5 g/kg/d L-Cit or 5 g/kg/d hydrolyzed soy protein (Iso-N-control) for 16 or 32 weeks. Additionally, 4-month-old C57BL/6J mice were treated accordingly for 8 weeks. Markers of senescence, glucose tolerance, intestinal barrier function, and intestinal microbiota composition were analyzed in mice. L-Cit treatment significantly extended the lifespan of *C. elegans*. The significant increase in markers of senescence and signs of impaired glucose tolerance found in 16- and 20-month-old control mice was attenuated in L-Cit-fed mice, which was associated with protection from intestinal barrier dysfunction and a decrease in NO_2_^−^ levels in the small intestine, while no marked differences in intestinal microbiota composition were found when comparing age-matched groups. Our results suggest that pharmacological doses of L-Cit may have beneficial effects on lifespan in *C. elegans* and aging-associated decline in mice.

## 1. Introduction

Worldwide, the number of old aged individuals is steadily increasing, and it has been projected that by 2050, the number of individuals >60 years of age will outnumber those <15 years of age [1]. Furthermore, results of the global burden of disease report 2019 also suggest that older age may increase the odds of developing diseases like Alzheimer’s disease and metabolic disease ([2]; for an overview, see [3,4]) and that the increases in life expectancy most of the time are not translated into an extended period of ‘healthy’ lifetime [5]. In addition, even for so-called ‘healthy’ aging, studies have shown that a healthy aging process is afflicted with functional impairments of cells, tissues, and organs, which finally lead to death [6,7]. Indeed, it has been suggested that aging should be regarded as a state of low-grade, sub-clinical inflammation, also called ‘inflammaging’, which develops in the absence of triggers like infections (for overview, see [8]) and even in healthy elderly individuals is going along with increased pro-inflammatory markers like plasminogen activator inhibitor (PAI-1) [9], interleukin 6 (IL6) and C-reactive protein (CRP) (for overview, see [10,11]). While it has been a key focus of many studies in recent years, it is still not fully understood how, when, and by what means aging intrinsically impairs cellular and tissue function and leads to ‘inflammaging’ and how this could be stopped or at least diminished.

In recent years, data from various species, including humans, has shown that aging and old age are associated with changes in the intestinal microbiota composition, including a decrease in α-diversity [12,13]. Furthermore, results of studies in rodents and some human studies suggest that along with the changes in intestinal microbiota composition, intestinal barrier function may also be impaired in older age [12,14,15]. For instance, it has been shown that in old mice, concentrations of tight junction proteins in small intestinal tissue are lower than in young animals [14]. In addition, we and others have shown that in the blood of healthy elderly humans and rodents, levels of bacterial endotoxin are higher than in young humans and rodents, respectively [14,16,17]. In support of the hypothesis that impairments of intestinal barrier function and a subsequent increased translocation of bacterial endotoxin may be critical in ‘inflammaging’, expression of Toll-like receptors (TLRs) has been shown to be induced with increasing age and that the loss of lipopolysaccharide binding protein (LBP) diminishes the aging-associated development of senescence and inflammation in liver tissue [16].

The results of several studies suggest that supplementation with the non-proteogenic amino acid L-Citrulline (L-Cit) may be beneficial in regard to several alterations associated with aging. For instance, it has been shown that a supplementation of L-Cit is associated with an improvement in physical performance in healthy elderly individuals while not affecting whole-body protein metabolism, blood flow, or microvascular circulation [18,19,20]. Furthermore, in rodents, it has been shown that supplementation with L-Cit may increase lean body mass and protect animals from lipid oxidation [21]. Breuillard et al. showed that a short-term supplementation of L-Cit diminished nitric oxide (NO) production in the peritoneal macrophages of elderly rats [22]. Results of our own studies but also those of others suggest that oral supplementation with L-Cit may affect intestinal microbiota composition and may have beneficial effects on intestinal barrier function in diseases of various etiologies [23,24,25]. However, whether a supplementation of L-Cit affects the length of a healthy lifespan and, if so, what are the molecular mechanisms related, e.g., alterations of the intestinal microbiota and barrier function, has not been clarified.

Starting from this background, in the present study, the effects of L-Cit on lifespan were assessed in Caenorhabditis elegans (*C. elegans*), whereas the effects of L-Cit on aging-associated decline and possible molecular mechanisms were determined in C57BL/6J mice.

## 2. Materials and Methods

### 2.1. Lifespan in C. elegans

N2 wildtype *C. elegans* and OP50 Escherichia coli (*E. coli*) were obtained from the Caenorhabditis Genetics Centre (CGC). *C. elegans* were cultured on nematode growth media (NGM) plates with OP50 standard food at 20 °C. To synchronize worms for lifespan experiments, L4 worms were placed on NGM plates with OP50 and maintained for 4 days. Eggs were collected, placed on new plates, and maintained for two additional days until worms were hatched. L1 larvae were transferred and kept on NGM plates ± 5 mM L-Cit at 20 °C. The concentration of L-Cit used in the *C. elegans* lifespan experiments was chosen based on a previous study of others assessing the effects of L-arginine (5 mM) discussed to exert at least in part similar physiological effects as L-Cit [26]. Controls were maintained on normal NGM plates without any nitrogen addition. Thereafter, the worms were transferred to fresh plates every day. By touching the posterior and anterior, it was assessed if worms were alive. Non-responding worms were counted as dead. Worms lost due to internal hatching were excluded from the survival analysis. Lifespan experiments were repeated three times and carried out in triplicates each time for each condition.

### 2.2. Animals and Treatment

**Figure 1:**
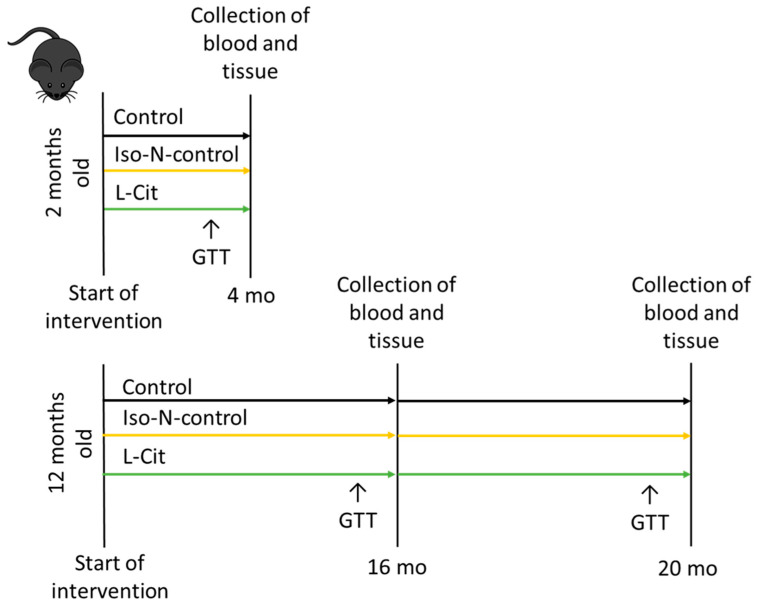
Overview of the design of the animal study. GTT, glucose tolerance test; Iso-N-control, hydrolyzed soy protein; L-Cit, L-Citrulline; 4 mo, 4 months; 16 mo, 16 months; 20 mo, 20 months.

The local Institutional Animal Care and Use Committee (IACUC) approved all animal protocols (Austrian Federal Ministry of Education, Science and Research, BMBWF-66.006/0014-V/3b/2019, 22 May 2019). The procedures were performed in a specific pathogen-free barrier facility accredited by the Association for Assessment and Accreditation of Laboratory Animal Care (AAALAC). Twelve-month-old male C57BL/6J mice (*n* = 8–10/group) were obtained from Janvier SAS France. An overview of the study design is given in Figure 1. Mice were fed a standard chow diet. In addition, some mice received drinking water enriched with 2.5 g L-Cit or 5 g hydrolyzed soy protein (Iso-N-control) per kg BW per day (Sigma Aldrich Chemie GmbH, Steinheim, Germany). Old mice were treated for 16 or 32 weeks, respectively. In addition, young C57BL/6J mice (*n* = 8/group, aged 2 months) fed standard chow received either plain drinking water or drinking water enriched with 2.5 g L-Cit or 5 g hydrolyzed soy protein (Iso-N-control) per kg BW per day for 8 weeks. Mice had free access to food and water. Consumptions of L-Cit and hydrolyzed soy protein water solutions were assessed several times per week, and L-Cit and Iso-N-control contents in water were adapted to water consumption. Food consumption and body weight were measured weekly. Two weeks prior to killing, a glucose tolerance test (GTT) was performed as previously explained [27]. In brief, after fasting mice for 6 h, fasting glucose was assessed, followed by an i.p. injection of glucose (2 g/kg BW). At the end of the study, mice were anesthetized with 100 mg/kg BW ketamine and 16 mg/kg BW xylazine (Richter Pharma AG, Wels, Austria) and killed by cervical dislocation. Mice were not fasted before killing. Blood was obtained from the portal vein, and pieces of intestine were snap-frozen in liquid nitrogen or used to assess intestinal permeability in the small intestine.

### 2.3. Measurement of Intestinal Permeability

Intestinal permeability of the small intestine was assessed immediately after tissue collection using xylose permeation as a marker, as described in detail before [28]. In the 4-month-old Iso-N-control group, some samples were lost due to technical issues when tissue was prepared so that xylose permeation could only be determined in *n* = 4.

### 2.4. Detection of Nitrite (NO_2_^−^)

Concentrations of nitrite in small intestinal tissue were assessed using a commercially available Griess reagent kit according to the manufacturer (Promega, Madison, WI, USA) and as previously described [28].

### 2.5. Western Blot

Plasma samples were separated on SDS-PAGE and transferred to PVDF membranes (Bio-Rad Laboratories, Hercules, CA, USA). Membranes were incubated with primary antibodies for p16 (Biorbyt Ltd., Cambridge, UK), intestinal fatty acid binding protein (I-FABP, Abcam, Cambridge, UK), IL6 (Santa Cruz Biotechnology, Inc., Heidelberg, Germany), and CRP (Santa Cruz Biotechnology, Inc., Heidelberg, Germany), and corresponding HRP-linked secondary antibodies. P16 and CRP proteins were detected on the same membrane. Protein bands were visualized with the Super Signal West Dura kit (Thermo Fisher Scientific, Waltham, MA, USA), and densitometrical analysis was carried out using the ChemiDoc XRS System with Image Lab software (Image Lab 6.1, Bio-Rad Laboratories, Hercules, CA, USA) and normalized to ponceau staining as detailed before [29,30].

### 2.6. Measurement of Total PAI-1

Concentrations of total PAI-1 protein in plasma were measured using a commercially available ELISA assay (LOXO GmbH, Dossenheim, Germany).

### 2.7. Measurement of TLR2 and TLR4 Ligands

Determination of TLR2 and TLR4 ligand levels in portal plasma was performed using a commercially available SEAP reporter HEK293 cell assay (InvivoGen, Toulouse, France) as described in detail before [31].

### 2.8. Microbiota Analysis

For microbiota analysis, total DNA was isolated from rinsed small intestines using Trizol reagent (Sigma Aldrich, Darmstadt, Germany) as described in detail before [32]. Quality and DNA yield were not sufficient for further analysis in samples of 2 young and 3 old mice in the control group, 3 young and 3 old mice in the Iso-N-group, and 4 young mice in the L-Cit group. The libraries were standardized and purified using the SequalPrep Normalization Kit (Invitrogen Inc., Carlsbad, CA, USA) and sequenced employing 250 bp paired-end sequencing chemistry on an Illumina MiSeq platform, as also detailed before [32]. Raw reads were quality filtered, assembled, and aligned against the database Silva version 132 using the MOTHUR pipeline [33]. Operational taxonomic units (OTU) were clustered at 97%. Singletons and low abundance reads (<0.0001%) were removed. Sequences were submitted to the European Nucleotide Archive under the accession number PRJEB65871.

### 2.9. Statistical Analysis

For statistical analysis, PRISM (Version 7.03, GraphPad Software, Inc., Boston, MA, USA) and R (version R4.2.2) were used. For data analysis of tissue and blood samples obtained in the mouse study, outliers were removed using Grubb’s outlier test. The data were tested for normal distribution using the Shapiro-Wilk normality test. A student *t*-test was used to compare two normally distributed groups. In case data were not normally distributed, data were log-transformed. When comparing more than two groups within one treatment group, e.g., mice of different ages fed L-Cit or Iso-N-fed mice or naïve controls, a one-way analysis of variance (ANOVA) followed by Tukey’s post hoc test was performed. No comparisons were performed between treatment groups.

The life span of *C. elegans* experiments was analyzed using Kaplan-Meier statistics. For analyzing microbiota composition, sample reads were normalized, and a sample-similarity matrix was generated utilizing the Bray-Curtis similarity coefficient in Primer 7 [34]. Differences in microbial community structure linked to age and dietary treatment were identified using permutational analysis of variance (PERMANOVA), and *p* ≤ 0.05 was considered significantly different. Visual community structure ordination was carried out through a non-metric multidimensional scaling plot (NMDS). Shannon’s Diversity Index was used to measure bacterial diversity. All data are shown as means ± standard error of the means (SEM). A statistical difference was defined as *p* ≤ 0.05.

## 3. Results

### 3.1. Effect of L-Cit on Lifespan in C. elegans

**Figure 2:**
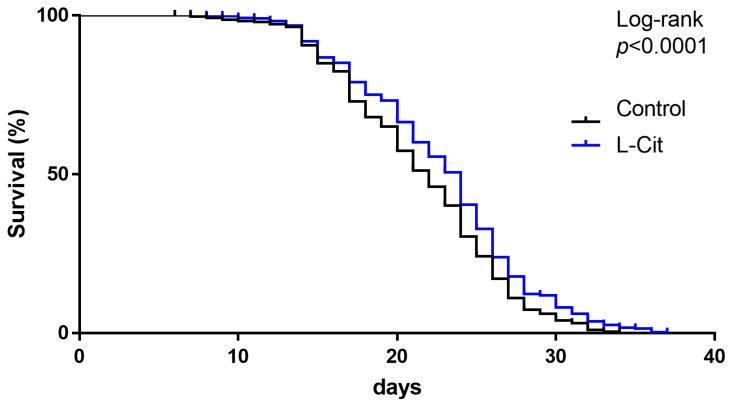
Effect of L-Cit on lifespan in *C. elegans*. Survival curve of the lifespan experiment of *C. elegans* treated with ±5 mM L-Cit. Kaplan-Meier survival analysis with the log-rank test. *C. elegans*, Caenorhabditis elegans; L-Cit, L-Citrulline.

As shown in Figure 2, the lifespan of worms kept on plates enriched with L-Cit after synchronization was significantly longer than that of naïve controls. Indeed, ~50% of worms kept under naïve conditions are still alive after 20 days, whereas on plates enriched with L-Cit, ~50% of worms were still alive after 23 days.

### 3.2. Effect of L-Cit on Markers of Senescence in C57BL/6J Mice

To determine if a supplementation of L-Cit also affects aging in mammals, young and middle-aged mice were fed a standard chow with plain drinking water (=naïve controls), a drinking water either fortified with 2.5 g L-Cit or 5 g hydrolyzed soy protein (Iso-N-control) per kg BW per day for 2 months (young animals) or 4 and 8 months (middle-aged mice), respectively. Within the control and Iso-N-control groups, absolute body weight and weight gain were significantly higher in 16- and 20-month-old mice than in 4-month-old mice, whereas in L-Cit-fed mice, only 20-month-old mice were heavier than 4-month-old mice (Table 1).

**Figure 3:**
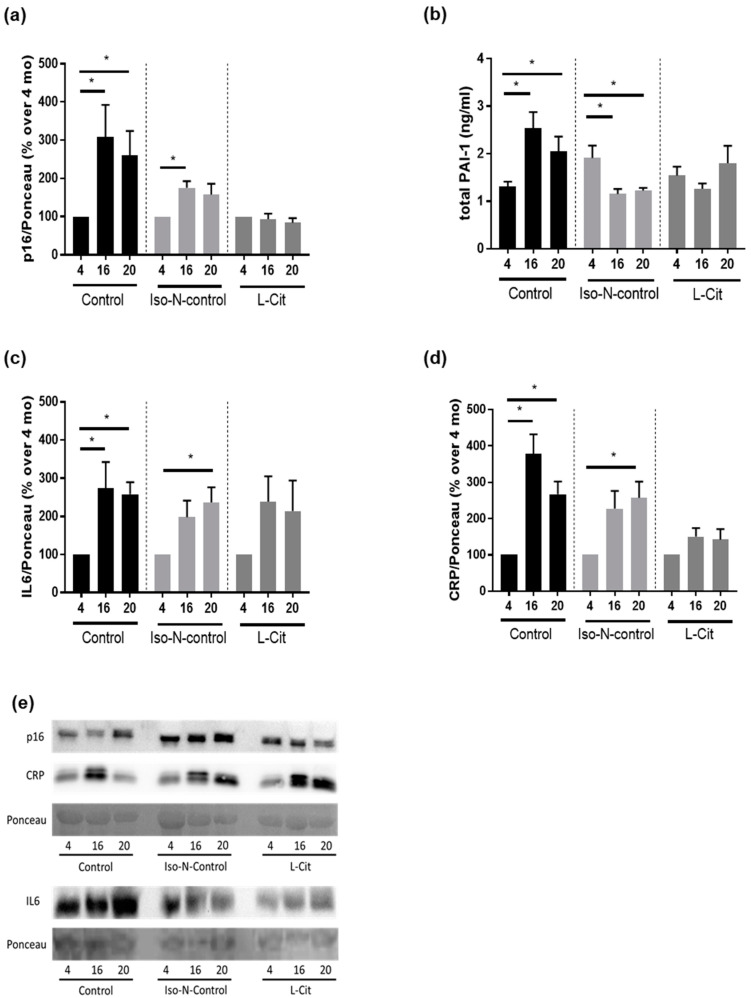
Effect of enriching drinking water with L-Cit or hydrolyzed soy protein (=Iso-N-control) on markers of senescence in 4-, 16-, and 20-month-old C57BL/6J mice. (**a**) p16 protein concentration in the western blot of plasma; (**b**) total PAI-1 concentration in plasma; (**c**) IL6 concentration in the western blot of plasma; (**d**) CRP concentration in the western blot of plasma; and (**e**) representative images of p16, CRP, and IL6 and corresponding Ponceau images. Representative images of full-size membranes of western blots are shown in Supplemental Figures S1–S5. Data are presented as means ± SEM, * *p* ≤ 0.05. CRP, C-reactive protein; IL6, interleukin 6; Iso-N-control, hydrolyzed soy protein; L-Cit, L-Citrulline; PAI-1, plasminogen activator inhibitor-1.

In naïve controls, p16 protein levels in plasma were significantly higher in both 16- and 20-month-old naïve control mice compared to the 4-month-old animals. In the plasma of Iso-N-fed mice, p16 protein levels were also significantly and by trend higher (*p* = 0.1) in the plasma of 16- and 20-month-old mice, respectively, compared to 4-month-old mice. Similar differences in plasma p16 protein levels were not found in mice fed drinking water enriched with L-Cit (Figure 3a and Supplemental Figures S1 and S2). Somewhat in line with these findings, protein levels of total PAI-1, also considered to be a marker of senescence [35], were also higher in 16- and 20-month-old naïve control mice compared to 4-month-old control animals. Similar differences were not found when comparing Iso-N-fed age groups (Figure 3b). Rather, in 16- and 20-month-old Iso-N-fed mice, PAI-1 protein levels in plasma were significantly lower than in 4-month-old Iso-N-fed animals. No differences were found when comparing PAI-1 plasma levels between L-Cit-fed age groups (Figure 3b). To further determine if the supplementation of L-Cit affected ‘inflammaging’, we next determined IL6 and CRP protein levels in plasma. Both CRP and IL6 protein concentrations in plasma were significantly and by trend higher in 16- and 20-month-old naïve controls and Iso-N-fed mice when compared to the respective 4-month-old groups (IL6: control mice: 4 vs. 16 months and 4 vs. 20 months *p* < 0.05, Iso-N-fed mice: 4 vs. 16 months *p* = 0.06 and 4 vs. 20 months *p* < 0.05; CRP: control mice: 4 vs. 16 months and 4 vs. 20 months *p* < 0.05, Iso-N-fed mice: 4 vs. 16 months *p* = 0.06 and 4 vs. 20 months *p* < 0.05). In contrast, in L-Cit-fed mice, neither CRP nor IL6 levels in plasma differ among age groups (Figure 3c,d, and Supplemental Figures S2–S5).

### 3.3. Effect of L-Cit on Markers of Glucose Metabolism

**Figure 4:**
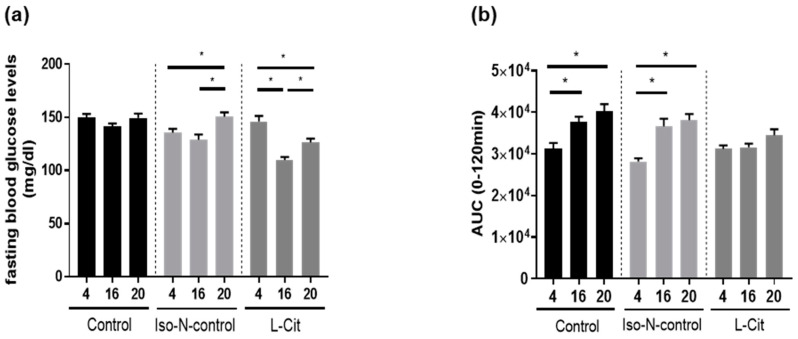
Effect of enriching drinking water with L-Cit or hydrolyzed soy protein (=Iso-N-control) on markers of glucose metabolism in 4-, 16-, and 20-month-old C57BL/6J mice. (**a**) Fasting blood glucose and (**b**) quantitative analysis of the area under the curve (AUC) of GTT. Data are presented as means ± SEM, * *p* ≤ 0.05. Iso-N-control, hydrolyzed soy protein; L-Cit, L-Citrulline; GTT, glucose tolerance test.

As aging has been shown not only to be associated with elevated senescence markers but also impairments of glucose tolerance and metabolism [36], we also compared fasting glucose levels and areas under the curve (AUC) of GTT. Blood glucose curves are shown in Supplemental Figure S6a. In naïve controls, fasting glucose levels were similar between age groups, whereas in 20-month-old Iso-N-fed mice, fasting glucose levels were significantly higher than in 4- and 16-month-old Iso-N-fed mice. In mice receiving L-Cit in drinking water, fasting glucose levels were significantly lower in 16- and 20-month-old mice compared to 4-month-old mice. Furthermore, when comparing the fasting glucose levels of 16- and 20-month-old L-Cit-fed mice, fasting glucose levels were found to be significantly higher in 20-month-old mice compared to 16-month-old mice (Figure 4a). Somewhat contrasting these findings, when analyzing the AUC of GTT between 0 and 120 min, the AUC of GTT was significantly higher in 16- and 20-month-old naïve control mice when compared to 4-month-old naïve control animals. Differences were also found when comparing the AUC of GTT in Iso-N-fed mice. In contrast, the AUC of GTT was similar between age groups in mice receiving L-Cit supplementation (Figure 4b). In addition, the AUC of GTT was analyzed between 0 and 30 min (see Supplemental Figure S6b). Somewhat in line with the findings of the analysis of the AUC of GTT for 0–120 min, the AUC of GTT between 0–30 min was significantly higher when comparing 4- and 20-month-old naïve control mice. No differences were found between young and 16-month-old naïve control mice. In addition, the AUC of GTT between 0 and 30 min was significantly higher in 16- and 20-month-old Iso-N-fed mice compared to their respective young mice, whereas in L-Cit-fed mice, the AUC of GTT between 0 and 30 min was similar between aging groups (see Supplemental Figure S6b).

As differences were most prominent when comparing 4- and 20-month-old mice, in the remaining analysis of tissue and plasma, we focused on the analysis of 4- and 20-month-old mice.

### 3.4. Effect of L-Cit on Intestinal Microbiota Composition in Small Intestines

**Figure 5:**
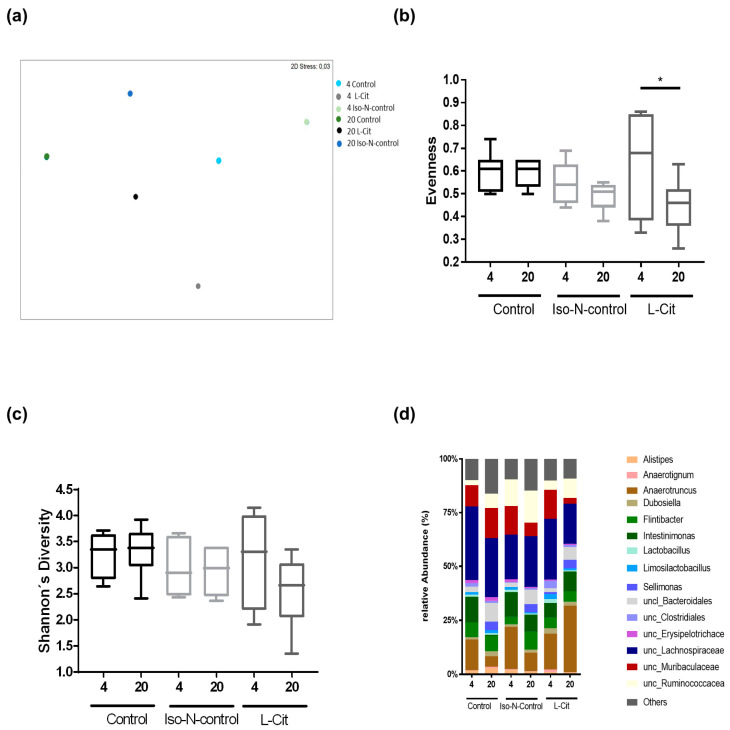
Effect of enriching drinking water with L-Cit or hydrolyzed soy protein (=Iso-N-control) on intestinal microbiota composition in 4- and 20-month-old C57BL/6J mice. (**a**) NMDS representing the centroids from the microbial communities in the small intestine; (**b**) species evenness; (**c**) Shannon’s diversity of the small intestine; as well as (**d**) the relative abundance of the most prevalent bacterial genera. Data are presented as means ± SEM, * *p* ≤ 0.05. Iso-N-control, hydrolyzed soy protein; L-Cit, L-Citrulline; NMDS, non-metric multidimensional scaling plot.

As it has been proposed in several studies of our working group that alterations of intestinal microbiota composition and intestinal barrier dysfunction in small intestine are afflicted with aging-associated decline and ‘inflammaging’ [12,37], and that L-Cit when being supplemented in pharmacological doses may affect both intestinal microbiota composition and barrier function in small intestine [38], we next determined if the beneficial effects of L-Cit on aging may be associated with changes in intestinal microbiota composition and/or barrier function in small intestine. As expected, microbial communities differed significantly between age groups; however, within age groups, there was only a trend for differences between the different treatments (*p* = 0.07) (Figure 5a). When assessing diversity evenness for microbial communities in the small intestine, no differences were found between 4- and 20-month-old naïve controls and 4- and 20-month-old Iso-N-fed mice, respectively. In contrast, diversity evenness was significantly different in L-Cit-fed groups, with 20-month-old L-Cit-fed mice showing a lower diversity (Figure 5b). Shannon’s diversity was similar among age groups, irrespective of treatment (Figure 5c). At the phylum level, the relative abundance of bacterial genera in the small intestine of 4- and 20-month-old mice is shown in Figure 5d. The relative abundance of *Sellimonas* was significantly higher in all 20-month-old mice compared to the respective 4-month-old mice, irrespective of additional treatments. In 20-month-old naïve mice, *Dubosiella* was significantly higher and *Intestinimonas* was significantly lower compared to 4-month-old naïve mice. In Iso-N-fed mice, the relative abundance of *Flintibacter* was significantly higher and that of *Anaerotignum* significantly lower in 20-month-old mice compared to their respective 4-month-old controls.

### 3.5. Effect of L-Cit on Markers of Intestinal Barrier Function

**Figure 6:**
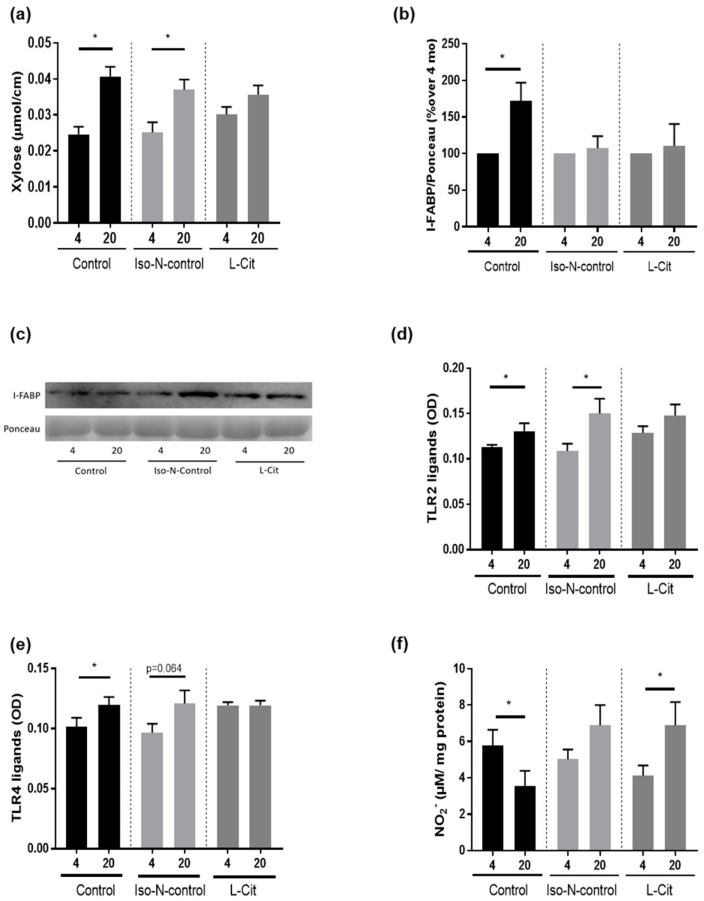
Effect of enriching drinking water with L-Cit or hydrolyzed soy protein (=Iso-N-control) on markers of intestinal barrier function in 4- and 20-month-old C57BL/6J mice. (**a**) Xylose permeation in the everted sacs of the small intestine. (**b**) I-FABP concentration in a western blot of plasma; (**c**) representative images of I-FABP and corresponding Ponceau image, ligands of (**d**) Toll-like receptor 2 (TLR2) and (**e**) TLR4 in plasma; as well as (**f**) NO_2_^−^ concentration in proximal small intestinal tissue. Representative images of full-size membranes of I-FABP Western blots are shown in Supplemental Figures S7 and S8. Data are presented as means ± SEM; * *p* ≤ 0.05. I-FABP, intestinal-fatty acid binding protein; Iso-N-control, hydrolyzed soy protein; L-Cit, L-Citrulline; NO_2_^−^, nitrite oxide.

Permeation of xylose in everted small intestinal tissue sacs was used as a marker of intestinal permeability and was found to be significantly higher in 20-month-old naïve control mice and Iso-N-fed 20-month-old mice when compared to their respective controls. Differences were not found when comparing 4- and 20-month-old L-Cit-fed mice (Figure 6a). In line with these findings, protein levels of I-FABP were also higher in 20-month-old naïve controls when compared to their respective controls, while being similar in 4- and 20-month-old L-Cit-fed mice (Figure 6b,c, and Supplemental Figures S7 and S8). No differences in plasma I-FABP protein levels were found between 4- and 20-month-old Iso-N-fed mice. Concentrations of TLR2 and TLR4 ligands (e.g., lipoteichoic acid (LTA) and lipopolysaccharide (LPS)) were also significantly higher in the portal plasma of 20-month-old naïve control mice compared to 4-month-old animals. Similar differences were also found when comparing TLR2 and TLR4 ligand concentrations in 4- and 20-month-old Iso-N-fed mice (TLR2: *p* < 0.05, TLR4 ligands: *p* = 0.064). In contrast, in mice fed L-Cit, TLR2 and TLR4 ligand concentrations in portal plasma were similar between age groups (Figure 6d,e). Furthermore, the concentration of NO_2_^−^ in small intestinal tissue was significantly lower in 20-month-old naïve controls compared to 4-month-old naïve controls, whereas in Iso-N-fed mice, NO_2_^−^ levels in small intestinal tissue did not differ between aging groups. In contrast, in 20-month-old L-Cit-fed mice, NO_2_^−^ levels in small intestinal tissue were significantly higher than in their respective young controls (Figure 6f).

## 4. Discussion

Aging is associated with a decline in cellular and organ function, subsequently altering overall physiological function. It has been proposed that enriching the diet with specific amino acids like branched-chain amino acids (L-leucine, L-isoleucine, and L-valine) or methionine may have adverse effects on longevity and metabolism (for an overview, see [39]), while supplementing other amino acids like L-arginine is discussed as having positive effects on longevity, probably due to its role as a precursor of NO [40]. However, results are mixed [41], and studies have reported adverse effects of a supplementation of L-arginine with respect to healthy aging [41]. The non-proteogenic amino acid L-Cit is known as the precursor of L-arginine in the urea cycle [42], but while having been shown to often mimic the effects of L-arginine and being herein even more efficient ([43] and for overview [44]), adverse effects seem to be lesser [45]. Here, employing *C. elegans* and C57BL/6 mice, we showed that the non-proteogenic amino acid L-Cit may extend lifespan in *C. elegans* and diminish the development of senescence and aging-related alterations in glucose tolerance in mice. Specifically, the lifespan of *C. elegans* was significantly longer when L-Cit was supplemented at pharmacological doses. These findings are in part in contrast to those of others assessing the effects of supplementing L-Cit on lifespan in *Drosophila* maintained under hypoxic conditions, where the addition of L-Cit to the medium was shown to dose-dependently shorten lifespan [46]. Differences between our results and those of others might have resulted from differences in species and study design (e.g., hypoxemia, starvation) [46,47] suggesting that the effects of L-Cit on lifespan may differ with respect to environmental or stress factors, e.g., the metabolic/disease status of the organisms. Indeed, in the study of Goron et al. (2019), employing mouse muscle cells as a model, it has been shown that supplementing L-Cit (5 mM) in stress conditions is associated with increased muscle synthesis resulting from a reallocation of mitochondrially generated ATP to protein synthesis [48]. Furthermore, Moinard et al. (2015) also showed a reduction in mortality in L-Cit-treated rats [21], supporting the hypothesis that a supplementation of L-Cit may have a positive effect on lifespan. However, when interpreting the data, it needs to be acknowledged that in the present study, no iso-nitrogenous control groups were carried along in the *C. elegans* lifespan experiments.

Somewhat in line with the findings for L-Cit on lifespan in *C. elegans*, plasma levels of markers of senescence and ‘inflammaging’ e.g., p16, PAI-1, IL6, and CRP were found to be markedly higher in old aged naïve controls. In contrast, these markers were also almost at the level of young L-Cit-fed mice in the plasma of 20-month-old L-Cit-fed mice. In Iso-N-fed mice, carried along as a control for the additional nitrogen intake derived through the supplementation of L-Cit, with the exception of total PAI-1 in plasma, these markers were also by trend or significantly higher in old-aged mice when compared to their respective young controls. The differential response of PAI-1 levels to aging might have resulted from a higher total PAI-1 protein level found in young animals, which might have resulted from a higher overall protein intake in mice when compared to naïve animals. Indeed, it has been shown that in humans, the composition of the diet, e.g., the fat, fiber, or protein content, may affect PAI-1 expression [49,50,51]. However, whether the addition of soy protein or other factors affected PAI-1 protein levels in plasma in the present study remains to be determined. Results of human studies have suggested that IL6 and CRP levels in blood are useful markers in the prediction of successful aging in the elderly and may even predict mortality [52]. The latter markers have been shown to be elevated in older aged individuals, and it was also shown that longer survival was associated with lower concentrations of both markers both in individuals with aging-related disease and disabilities as well as those successfully aging [52]. Similar results have also been reported for PAI-1 (for an overview, see [35]). Moreover, the results of studies suggest that p16 can act as a regulator of not only IL6 but also CRP [53,54]. However, whether L-Cit alters senescence and ‘inflammaging’, e.g., p16 or IL6, and CRP protein levels through direct or indirect mechanisms (see below), remains to be determined. In addition, in line with the findings for markers of senescence, the AUC of GTT was unchanged over time in mice receiving L-Cit in their drinking water while being significantly higher in older naïve controls and Iso-N-fed controls when compared to their respective young controls. Differences were only in part found when assessing fasting glucose levels. Indeed, fasting glucose levels were unaffected by age in naïve controls while being higher in older aged Iso-N-fed mice. The supplementation of L-Cit was associated with significantly lower fasting glucose levels in older-aged animals. It has been suggested by the results of others, too, that even in a setting of healthy aging, glucose tolerance is impaired in older aged mice [55]. The lack of differences in fasting glucose levels in naïve 20-month-old mice is in line with the findings of others showing that in older-aged C57BL/6 mice but also in humans, fasting glucose levels may be unchanged when compared to young animals while glucose tolerance is impaired, suggesting that a decline in glucose homeostasis is not an inevitable consequence of aging in mice [56]. A supplementation of L-Cit has been proposed to alter glucose metabolism before, e.g., in settings of obesity and type 2 diabetes [57,58], being thought to be related to alterations of NO synthesis.

Taken together, our results suggest that a supplementation of pharmacological doses of L-Cit may extend a healthy lifespan in *C. elegans* and may affect senescence and glucose metabolism in aging mice, further suggesting that in settings of healthy aging, a supplementation of L-Cit may have beneficial effects on aging-associated degenerative processes. However, it remains to be determined if similar effects are also found in humans and settings of ‘unhealthy’ aging, e.g., when metabolic or cognitive diseases are prevalent.

### The Protective Effects of L-Cit Are Associated with a Protection against the Aging-Associated Loss of Intestinal Integrity but Not with Changes of Intestinal Microbiota Composition

Results of several studies suggest that even healthy aging is associated with alterations of intestinal microbiota composition both in the small and large intestines ([12,59]; and for an overview, see [60]) and impairments of the intestinal barrier function [14,61]. In the present study, we focused on the composition of intestinal microbiota in the small intestine, which differed significantly between young and old mice both at the level of genus and at the level of some species, being in line with previous findings of us and others [12,62]. It has been proposed that amino acids like L-Cit can be metabolized by intestinal microbiota and that supplementation may be associated with changes in mucosal microbiota composition in the large intestine [25], while effects on intestinal barrier function were reported mainly for the small intestine (for an overview, see [38]). In the present study, evenness was significantly lower in the small intestine of 20-month-old mice treated with L-Cit, while differences were not found when comparing the age groups of naïve and Iso-N-fed mice. However, no differences were observed at the level of species. Differences between the results of the present study and those of others might have resulted from differences in species (rats vs. mice) and tissue studied (small intestine vs. large intestine) [25]. Indeed, in previous studies of our own group supplementing L-Cit to mice fed a fructose-, fat-, and cholesterol-rich diet, we also found limited effects of oral L-Cit supplementation on intestinal microbiota composition in the small intestine when compared to mice fed the diet without the supplement [28].

It has also been shown that L-Cit, when supplemented at pharmacological doses, may interfere with the development of intestinal barrier dysfunction in diseases of various etiologies (e.g., metabolic dysfunction-associated steatotic liver disease, intestinal obstruction) [28,63]. In the present study, intestinal permeability, as determined by measuring xylose permeation in small intestinal everted tissue sacs, was also significantly higher in 20-month-old naïve controls and Iso-N-fed mice, while not differing between young and old-aged mice receiving L-Cit. These findings are in line with previous findings from our group, showing that intestinal permeability is higher in older aged mice than in young animals [14]. In line with these findings, I-FABP protein levels were also higher in the plasma of old-aged naïve controls while not differing between young and old-aged mice receiving the L-Cit supplementation. The lack of difference in I-FABP protein levels in the Iso-N-groups might have been related to the additional protein intake in this group. It has been shown before in human studies that a supplementation of protein (bovine colostrum) may attenuate the increase of I-FABP in blood found after exercise [64]. Still, the apparent discrepancy between the findings for I-FABP protein levels and xylose permeation remains to be determined. However, further supporting the finding that the oral supplementation of L-Cit attenuated aging-associated decline of intestinal barrier function, the concentration of TLR2 and TLR4 ligands in portal plasma was also not altered in 20-month-old mice treated with L-Cit compared to young animals, while being higher in the portal plasma of old-aged naïve mice and animals fed drinking water enriched with protein.

Results of our own group have previously suggested that intestinal NO-homeostasis may be altered in aging and that these alterations, e.g., lower NO concentrations and higher arginase activity, may be associated with impairments of intestinal barrier function in the small intestine [14]. Furthermore, L-Cit has been proposed to be an allosteric regulator of arginase activity, the counterplayer of nitric oxide synthase, and a NO precursor (for an overview, see [65]). It has also been shown that supplementation with L-Cit increases NO levels in the small intestine in mice, with endotoxemia being associated with improved microcirculation [66]. In the present study, NO_2_^−^ levels in the small intestinal tissue of naïve 20-month-old mice were lower than in their respective controls, whereas in Iso-N-fed 20-month-old mice, NO_2_^−^ levels were similar to those of their respective young controls. In contrast, NO_2_^−^ levels were markedly higher in the small intestinal tissue of 20-month-old mice treated with L-Cit. In line with these findings, we previously reported that inhibiting arginase activity in small intestines in aging mice by treating mice with norNOHA not only resulted in increased NO_2_^−^ formation in small intestinal tissue but also in an alleviation of intestinal barrier dysfunction in small intestines associated with aging and lower p16 levels in liver tissue [14].

## 5. Conclusions

Taken together, the results of the present study further bolster the hypothesis that a supplementation of pharmacological doses of L-Cit may have beneficial effects on aging-associated decline and may even extend a healthy lifespan. Our results also suggest that the beneficial effects of L-Cit may, at least in part, be related to an interaction of this non-proteogenic amino acid with intestinal barrier function. Further studies are needed to determine (1) the molecular mechanisms underlying the beneficial effects of L-Cit on intestinal barrier function in aging, (2) if these effects are persistent over a longer period of time and thereby add to an extension of healthy lifespan in higher organisms, and (3) if a treatment with L-Cit may also have beneficial effects in aging healthy but even more so in diseased humans.

## Figures and Tables

**Table 1:** Effect of enriching drinking water with L-Cit or hydrolyzed soy protein (=Iso-N-control) on end body weight and weight gain in 4-, 16-, and 20-month-old C57BL/6J mice.

	Groups	Age (Months)		
		4	16	20
Absolute end body weight (g)	Control	29.2 ± 0.6	34.3 ± 1.0 *	33.8 ± 0.7 *
	Iso-N-control	29.5 ± 0.6	34.6 ± 0.8 *	36.2 ± 1.3 *
	L-Cit	30.3 ± 0.7	32.5 ± 1.0	34.5 ± 1.2 *
Absolute weight gain (g)	Control	5.7 ± 0.3	2.2 ± 0.6 *	2.0 ± 0.7 *
	Iso-N-control	5.9 ± 0.5	2.0 ± 0.2 *	3.0 ± 0.4 *
	L-Cit	6.3 ± 0.5	1.6 ± 0.2 *	3.5 ± 0.7 *

Data are shown as means ± SEM. * *p* ≤ 0.05 compared to respective 4-month-old mice in the same feeding group. Iso-N-control, hydrolyzed soy protein; L-Cit, L-Citrulline.

## Data Availability

Data will be made available on reasonable request. Sequencing data were submitted to the European Nucleotide Archive under the accession number PRJEB65871.

## Supplementary Materials

The following supporting information can be downloaded at: https://www.mdpi.com/article/10.3390/biom13111579/s1, Figure S1: Uncropped blot and representative picture of p16 in the western blot of plasma in Figure 3e. Figure S2: Ponceau and representative picture of Ponceau of p16 and CRP in Figure 3e. Figure S3: Uncropped blot and representative picture of CRP in the western blot of plasma in Figure 3e. Figure S4: Uncropped blot and representative picture of IL6 in the western blot of plasma in Figure 3e. Figure S5: Ponceau and representative picture of Ponceau of IL6 in Figure 3e. Figure S6: Effect of enriching drinking water with L-Cit or hydrolyzed soy protein (=Iso-N-control) on blood glucose levels in 4-, 16-, and 20-month-old C57BL/6J mice. Figure S7: Uncropped blot and representative picture of I-FABP in the western blot of plasma in Figure 6c. Figure S8: Ponceau and representative picture of Ponceau of I-FABP in Figure 6c.
