# Supplementary material for: Supplementing L-Citrulline Can Extend Lifespan in C. elegans and Attenuate the Development of Aging-Related Impairments of Glucose Tolerance and Intestinal Barrier in Mice
Source: Biomolecules. 2023 Oct 26;13(11):1579. doi: 10.3390/biom13111579 (PMC10669166; doi:10.3390/biom13111579)
Supplement: Supplementary file 1 [file biomolecules-13-01579-s001.zip › biomolecules-2640887-supplementary.pdf]

Supplementary Materials

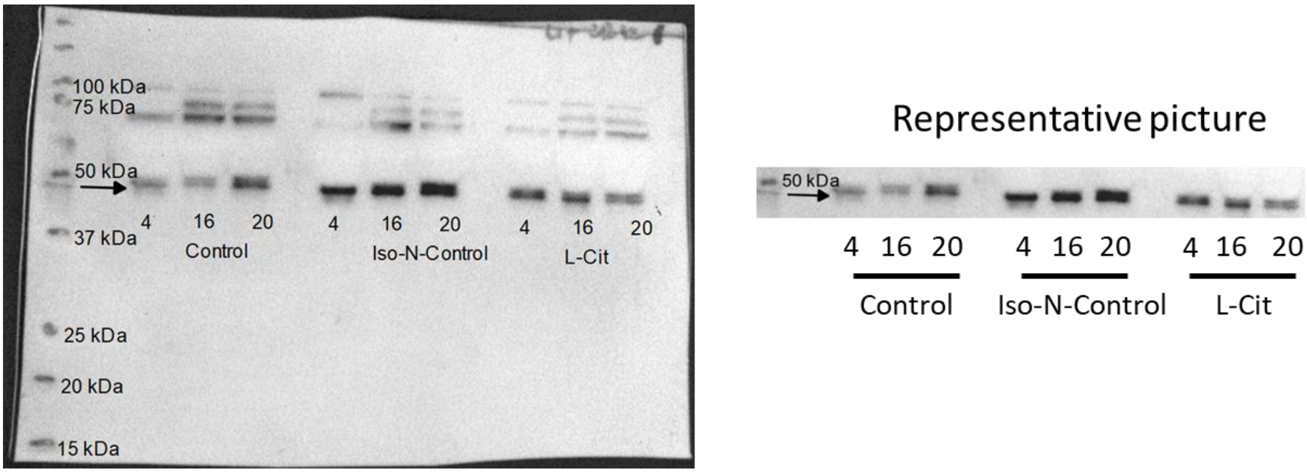

Figure S1. Uncropped blot and representative picture of p16 in western blot of plasma in Figure 3e. L-Cit, L-Citrulline; Iso-N-control, hydrolyzed soy protein.

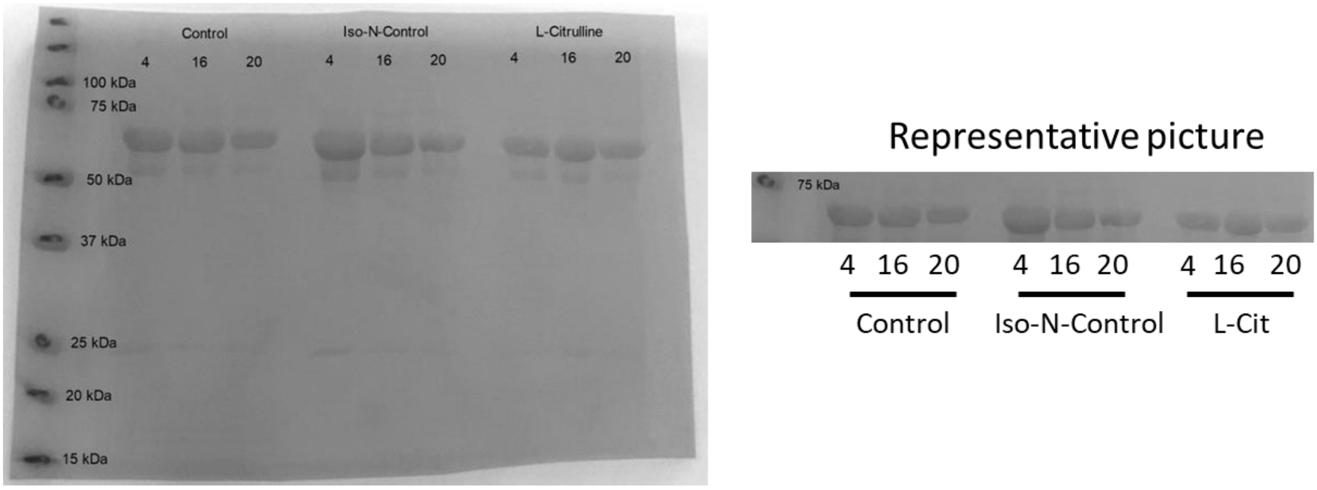

Figure S2. Ponceau and representative picture of Ponceau of p16 and CRP in Figure 3e. L-Cit, L-Citrulline; Iso-N-control, hydrolyzed soy protein.

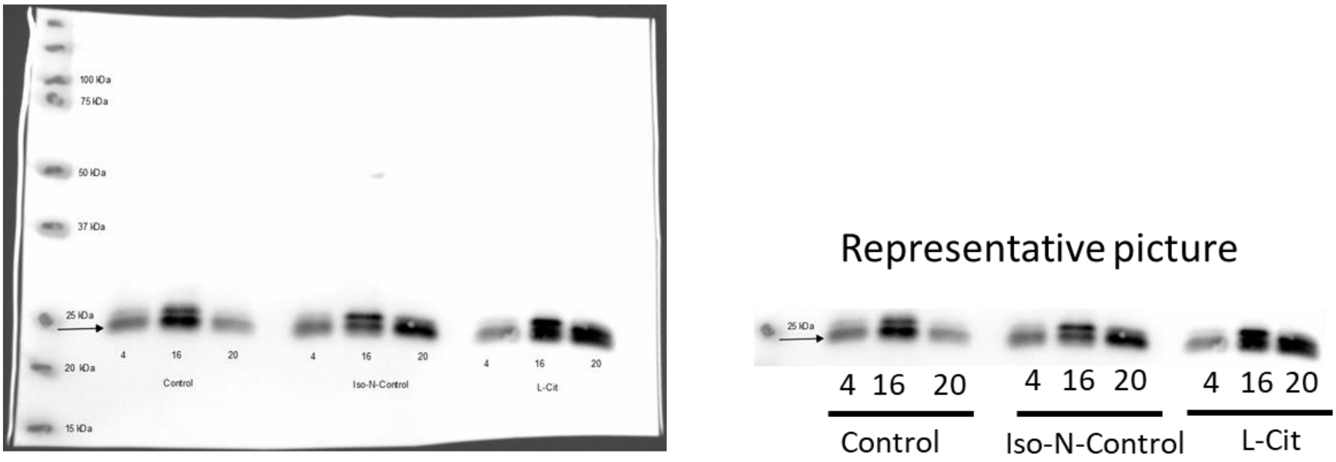

Figure S3. Uncropped blot and representative picture of CRP in western blot of plasma in Figure 3e. L-Cit, L-Citrulline; Iso-N-control, hydrolyzed soy protein.

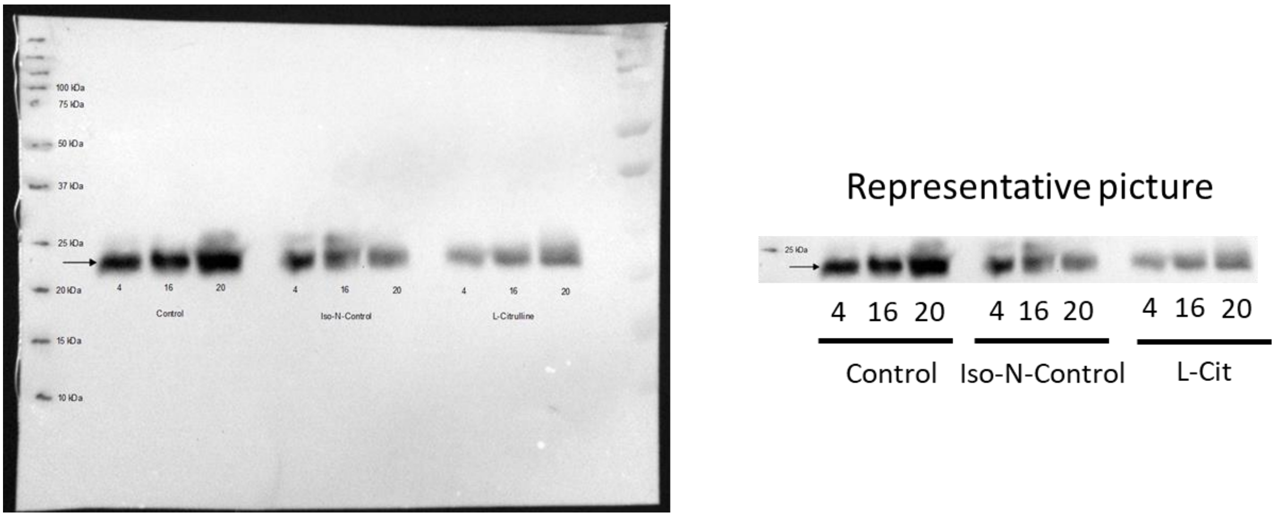

Figure S4. Uncropped blot and representative picture of IL6 in western blot of plasma in Figure 3e. L-Cit, L-Citrulline; Iso-N-control, hydrolyzed soy protein.

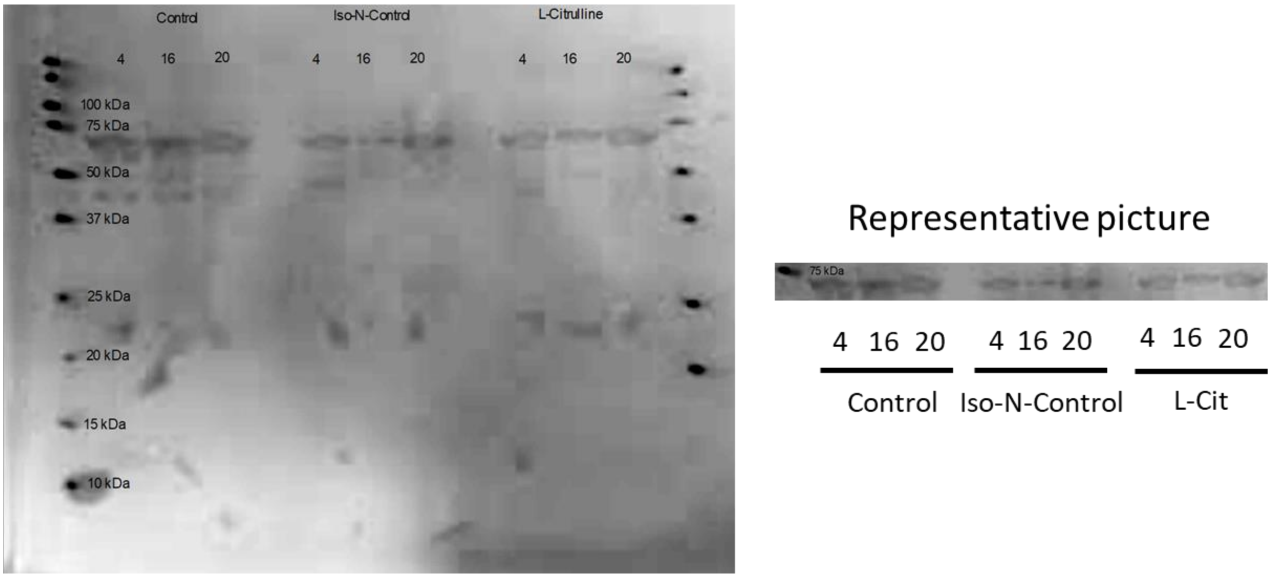

Figure S5. Ponceau and representative picture of Ponceau of IL6 in Figure 3e. L-Cit, L-Citrulline; Iso-N-control, hydrolyzed soy protein.

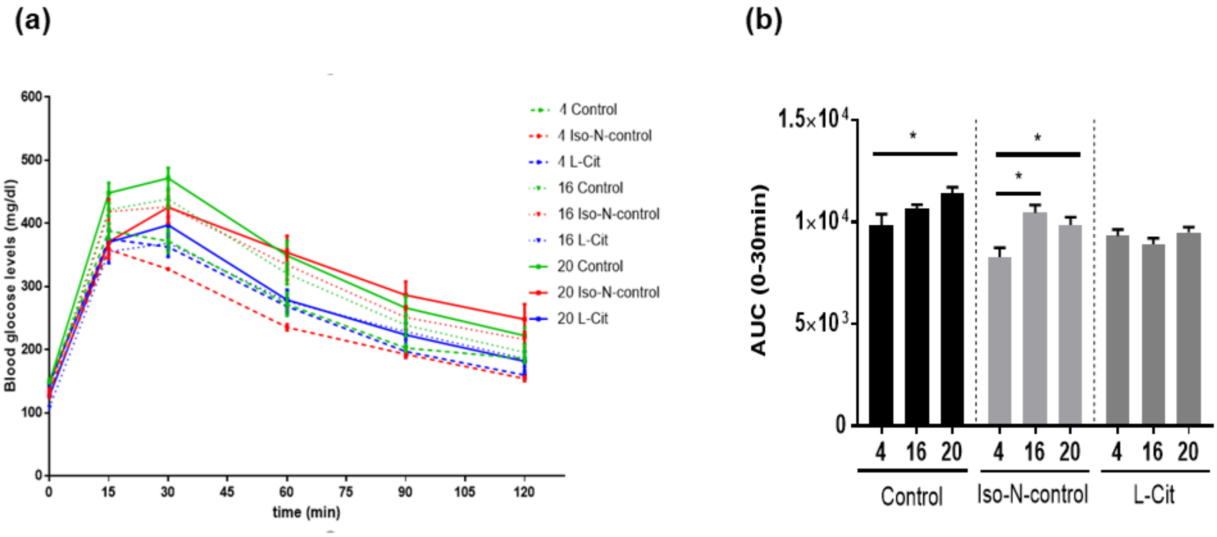

Figure S6. Effect of enriching drinking water with L-Cit or hydrolyzed soy protein (=Iso-N-control) on blood glucose levels in 4-, 16- and 20-months old C57BL/6J mice. (a) Blood glucose levels of GTT and (b) quantitative analysis of area under the curve (AUC) during GTT (0-30 min). Data are presented as means ± SEM, * $p \leq 0.05$. Iso-N-control, hydrolyzed soy protein; L-Cit, L-Citrulline; GTT, glucose-tolerance-test.

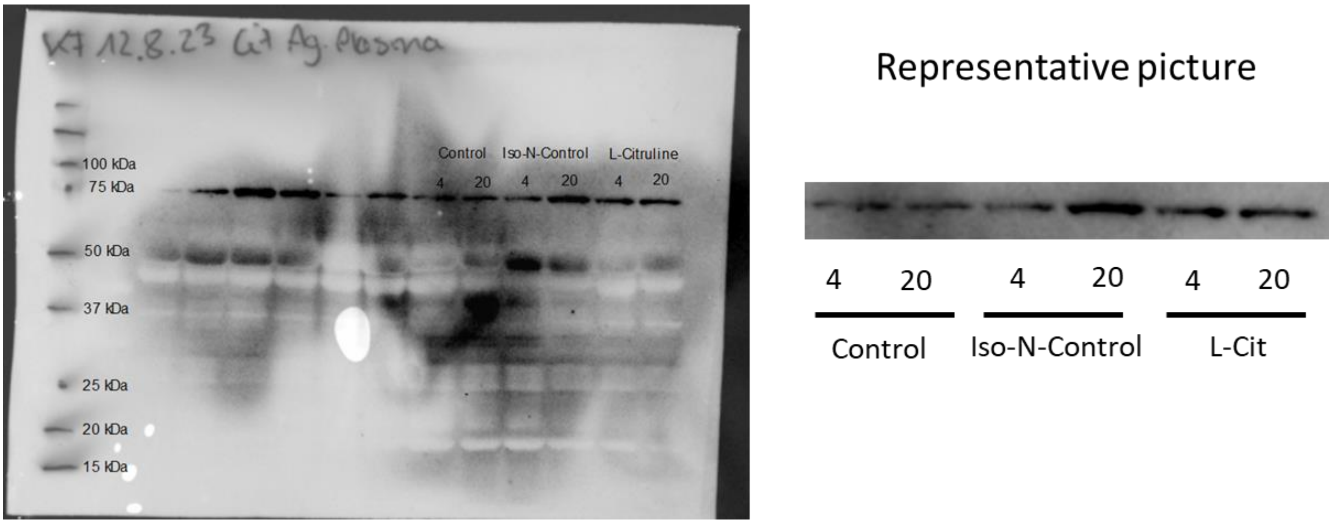

Figure S7. Uncropped blot and representative picture of I-FABP in western blot of plasma in Figure 6c. L-Cit, L-Citrulline; Iso-N-control, hydrolyzed soy protein.

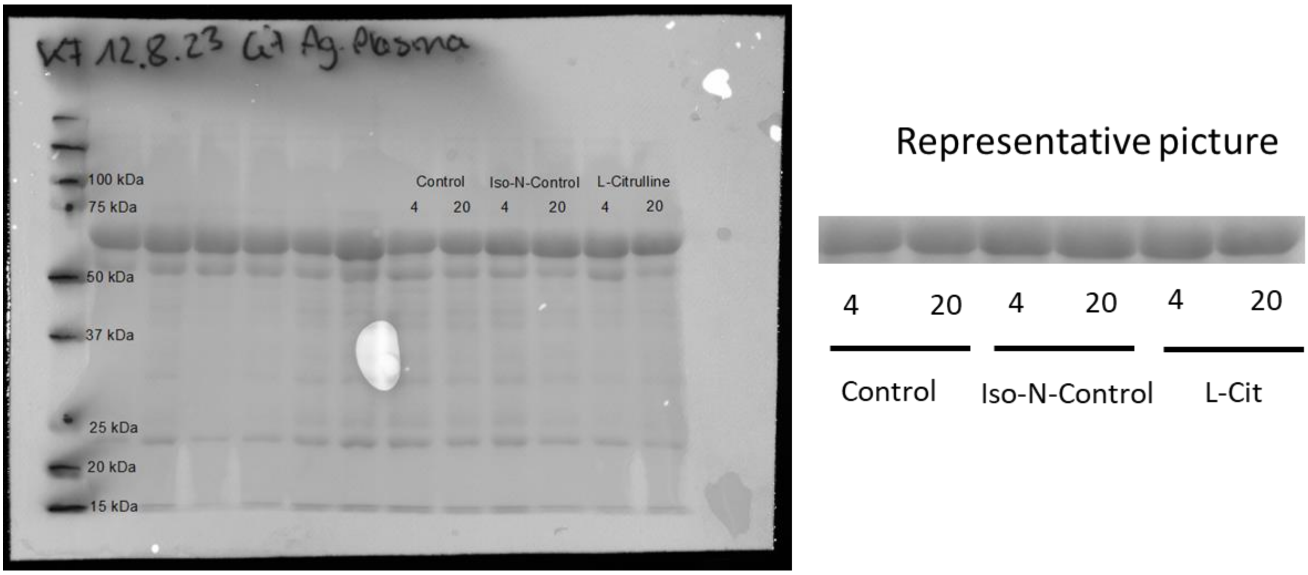

Figure S8. Ponceau and representative picture of Ponceau of I-FABP in Figure 6c. L-Cit, L-Citrulline; Iso-N-control, hydrolyzed soy protein.
